# Alginate Hydrogels Coated with Chitosan for Wound Dressing

**DOI:** 10.3390/md13052890

**Published:** 2015-05-11

**Authors:** Maria Cristina Straccia, Giovanna Gomez d’Ayala, Ida Romano, Adriana Oliva, Paola Laurienzo

**Affiliations:** 1 Institute for Polymers, Composites and Biomaterials (IPCB), CNR, via Campi Flegrei 34, Pozzuoli 80078, Italy; E-Mails: mariacristina.straccia@ictp.cnr.it (M.C.S.); giovanna.gomez@ictp.cnr.it (G.G.A.); 2 Institute of Biomolecular Chemistry, CNR, via Campi Flegrei 34, Pozzuoli 80078, Italy; E-Mail: iromano@icb.cnr.it; 3 Department of Biochemistry, Biophysics and General Pathology, Second University of Naples, via L. De Crecchio 7, Naples 80138, Italy; E-Mail: adriana.oliva@unina2.it

**Keywords:** chitosan hydrochloride, alginate, hydrogels, antibacterial activity, sustained release, wound dressing

## Abstract

In this work, a coating of chitosan onto alginate hydrogels was realized using the water-soluble hydrochloride form of chitosan (CH-Cl), with the dual purpose of imparting antibacterial activity and delaying the release of hydrophilic molecules from the alginate matrix. Alginate hydrogels with different calcium contents were prepared by the internal setting method and coated by immersion in a CH-Cl solution. Structural analysis by cryo-scanning electron microscopy was carried out to highlight morphological alterations due to the coating layer. Tests *in vitro* with human mesenchymal stromal cells (MSC) were assessed to check the absence of toxicity of CH-Cl. Swelling, stability in physiological solution and release characteristics using rhodamine B as the hydrophilic model drug were compared to those of relative uncoated hydrogels. Finally, antibacterial activity against *Escherichia coli* was tested. Results show that alginate hydrogels coated with chitosan hydrochloride described here can be proposed as a novel medicated dressing by associating intrinsic antimicrobial activity with improved sustained release characteristics.

## 1. Introduction

The local treatment of wounds is crucial to prevent infections, to control the removal of exudates and to create a moist environment to allow for skin healing [1]. Research is nowadays increasingly oriented towards “bioactive dressings”. These dressings are made of materials that can play an active role in wound protection and healing. The first strategy consists of the application of skin substitutes, following a cell therapy approach [2]. A second strategy is the realization of “medicated dressings”, able to release biomolecules in a sustained manner and to perform functions other than passive protection [3].

Due to their characteristics, hydrogels find application in wound dressing, especially in cases where a conventional dressing can be difficult to apply, as deep and irregular lesions. Hydrogels based on natural polysaccharides are highly hydrophilic and sometimes sensitive to enzymatic degradation [4,5,6]. In particular, alginates (Alg) are known for their ability to crosslink under mild conditions through a series of divalent cations. Alginates are a family of popular biocompatible polysaccharides extracted from brown algae [7]. Their hydrogels, in combination with other biopolymers or active agents, are used as dressing for wounds and burns, as they help to maintain an optimum moisture environment and cool temperature [8,9,10]. Alginate is approved for healthcare and present on the market with several trademarks. Another merit of alginate hydrogels is that they can be easily and quickly prepared at the time of need starting from sterilized solutions in a sterile area, making them appealing from a galenic, as well as an industrial point of view.

Alginate beads and hydrogels are stable in acidic media, whereas they easily swell and disintegrate in alkaline media and normal saline solution [11], conditions similar to those found in wound exudates. As calcium ions are being released by the ion exchange with sodium in the medium, electrostatic repulsion between the carboxylate anions further accelerates the swelling and erosion of alginate gels [12]. Moreover, on account of short time release in alkaline and neutral media, alginate is not an ideal material for sustained release. There were many attempts to control the disintegration of alginate-based drug delivery systems (DDS) and extend drug release characteristics through coating with polycationic polymers, such as poly(l-lysine) [13] or chitosan [14]. In contrast to alginate, which is polyanionic in nature, chitosan is a polycationic polysaccharide, derived from chitin [15,16]. Due to its gel-forming properties, it has been also employed in the design of DDS [17,18]. Complexation of alginate with chitosan reduces the porosity of the alginate gel and decreases the leakage of the encapsulated drugs [19,20,21,22,23,24]. In the case of wound dressing, encapsulated drugs are mainly antibiotics able to prevent or combat infections or growth factors to accelerate the healing process.

Nowadays, there are many concerns about the prolonged use of antibiotics, as required in the case of chronic wounds. The rise of bacterial resistance to antibiotics is well documented, both in the scientific literature and in the popular press. The World Health Organization recently described antimicrobial resistance as “a problem so serious that it threatens the achievements of modern medicine” [25]. Another concern is the safety of silver and titanium nanoparticles, often used in wound dressing for their antimicrobial activity [26,27]. Due to the small size, nanoparticles are easily absorbed into biological tissues and may interact with mitochondria, inducing structural damages, specifically in the liver cells [28]. In this regard, the use of materials with intrinsic antibacterial activity, avoiding the release of nanoscopic particles, may represent a valid alternative. As the antibacterial properties of chitosan are well documented in the literature [29,30], coating of alginate hydrogels with chitosan looks to be a possible strategy to realize intrinsic antimicrobial dressings with sustained release characteristics. Anyway, the difficulty in realizing alginate-chitosan hydrogels derives from their different pH-dependent water solubilities. To overcome these drawbacks, several examples of the use of water-soluble chitosan derivatives are found in literature [31,32]. Nevertheless, chemical modification usually involves amines, with the loss of antibacterial activity.

In the present work, a coating of chitosan onto alginate hydrogels was realized using chitosan hydrochloride (CH-Cl), a protonated form of chitosan, with the dual purpose of imparting antibacterial activity and delaying the release of hydrophilic molecules from the alginate matrix. Chitosan hydrochloride was characterized by FTIR analysis. Alginate hydrogels with different calcium contents were prepared by the internal setting method and coated by immersion in a CH-Cl solution. Structural analysis by cryo-scanning electron microscopy permits highlighting the morphological alterations due to the coating layer. Tests *in vitro* with human mesenchymal stromal cells (MSC) were assessed to check the absence of toxicity of CH-Cl. Swelling, stability in physiological solution and release characteristics using rhodamine B as the hydrophilic model drug were compared to those of relative uncoated hydrogels. Finally, the antibacterial activity against *Escherichia coli* was tested.

## 2. Results and Discussion

### 2.1. Preparation of CH-Cl

**Figure 1:**
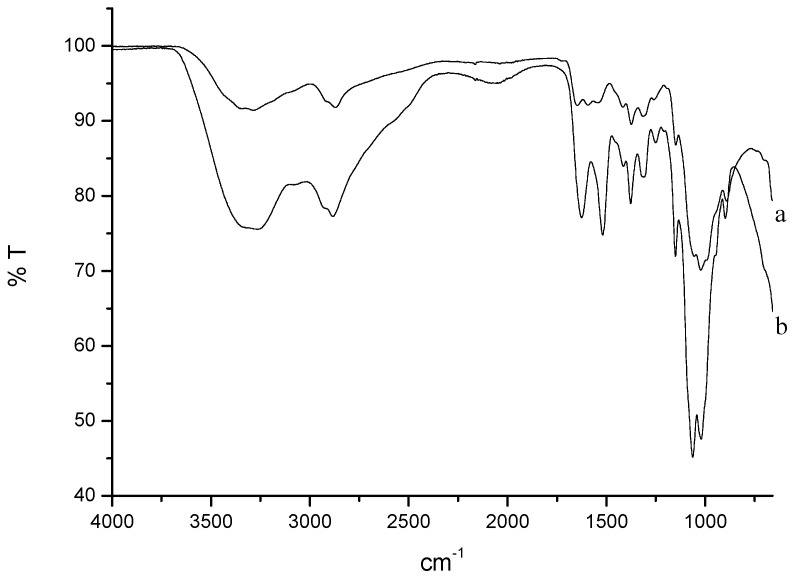
(**a**) FTIR spectrum of chitosan; (**b**) FTIR spectrum of chitosan hydrochloride (CH-Cl).

CH-Cl is a charged form of chitosan, completely soluble in acid-free water [33]. It is known from the literature that the methodology employed here results in highly pure samples, which preserve an identical degree of deacetylation [34]. FTIR spectra of chitosan and CH-Cl are shown in Figure 1. The appearance in the CH-Cl spectrum of typical bands of symmetric and asymmetric stretching of ammonium N-H (1624 and 1518 cm^−1^, respectively) is evidence of the protonation of free amines.

### 2.2. Preparation and Preliminary Characterization of Hydrogels

Alginate hydrogels were prepared via internal gelation, using CaCO_3_ as the calcium source. This methodology allows one to obtain highly regular and homogeneous gels through a slow release of calcium ions. Different CaCO_3_ and d-(+)-gluconate-δ-lactone (GDL) amounts were used, as reported in Table 1.

**Table 1:** Composition and codes of hydrogels. GDL, d-(+)-gluconate-δ-lactone; Alg, alginate.

CaCO_3_ (mmol)	GDL (mmol)	GDL (mL)	Alg/Ca^2+^ * (mol/mol)	GDL/CaCO_3_ (mol/mol)	Code
0.999	0.999	17.8	5.155	1	**Alg1**
0.999	1.998	35.6	5.155	2	**Alg2**
1.998	1.998	35.6	2.577	1	**Alg3**
1.998	3.997	71.2	2.577	2	**Alg4**

* The reported data refer to 1 g of alginate = 0.0051 mol of repeating units.

The intrinsic features of hydrogels are strictly dependent on the relative concentrations of calcium carbonate and GDL. In particular, the molar ratio between GDL and calcium carbonate is crucial to obtain a complete salt dissolution and to determine the final gel pH (acid or neutral) [35,36], whereas the molar ratio between alginate repeating units and calcium ions regulates the number of carboxylates engaged in ionic interactions and, therefore, the crosslinking density of the resulting gel. Such characteristics are important for the final properties of the gel and for the interactions of alginate with chitosan hydrochloride, as will be discussed. Two GDL/CaCO_3_ molar ratios were chosen, in order to obtain neutral or acid gels (1/1 and 2/1, respectively). Concerning the Alg/Ca^2+^ ratio, a value close to the stoichiometric one or its half (2.5/1 and 5/1) was investigated.

Coating of alginate hydrogels was achieved simply by immersion in a 1% (*w/w*) CH-Cl water solution. The dipping time (1 h) and CH-Cl concentration were established after several attempts. During immersion, shrinkage is observed for all hydrogels to a different extent. All coated hydrogels (c-Alg) retain transparency, a desirable feature for wound dressing to allow inspection of the injury bed. Furthermore, the hydrogels are harder and easier to handle upon coating.

Gelation time, bulk pH value and water content are reported in Table 2. Gelation time is mainly influenced by the calcium ion concentration, passing from a slow (around half an hour) to a fast (a few minutes) gelation when the amount of calcium carbonate is doubled. Of course, an excess of GDL (GDL/CaCO_3_ equal to two) further accelerates gelation as a consequence of two effects: faster calcium carbonate dissolution and partial acidification of carboxylate groups, which contribute to gelation via hydrogen bonding.

pH measurements evidence that hydrogels with a GDL/Ca^2+^ molar ratio equal to one (Alg1 and Alg3) are basic, suggesting that calcium carbonate is not completely neutralized. When an excess of GDL is used (Alg2 and Alg4), the pH is lowered until neutral. After coating, a decrease of bulk pH values is generally observed, likely due to diffusion of CH-Cl within the hydrogel during immersion. In particular, c-Alg2 and c-Alg4 show an acid pH value.

**Table 2:** Gelation times, pH values and water content. c, coated.

Sample	Gelation Time (min)	pH	Water Content (wt %)
**Alg1**	40 ± 4	8.34 ± 0.14	69.37 ± 1.32
**Alg2**	28 ± 3	6.49 ± 0.70	68.61 ± 1.15
**Alg3**	8 ± 1	7.24 ± 0.83	69.63 ± 0.32
**Alg4**	5 ± 1	7.12 ± 0.01	68.76 ± 0.84
**c-Alg1**	*	7.53 ± 0.54	69.42 ± 0.96
**c-Alg2**	*	5.19 ± 0.36	67.70 ± 0.82
**c-Alg3**	*	7.34 ± 0.81	65.94 ± 1.02
**c-Alg4**	*	5.10 ± 0.40	53.51 ± 0.64

* Gelation times are the same as the corresponding uncoated hydrogels.

The water content of Alg hydrogels is in line with what is reported in the literature for calcium alginate spray formulations [37]. Upon coating, a slight decrease is generally observed, likely due to the release of water associated with shrinking. This behavior is more pronounced in the case of c-Alg4, which visually shows greater shrinkage associated with volume reduction.

**Figure 2:**
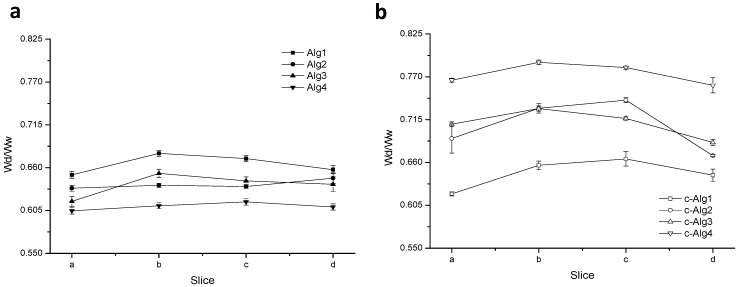
(**a**) Homogeneity profiles of Alg hydrogels; (**b**) homogeneity profiles of c-Alg hydrogels.

Figure 2 displays the gel homogeneity, estimated according to the literature [38]. The results show that, despite shrinking, the coating does not substantially influence the homogeneity of the gels.

### 2.3. Swelling

**Figure 3:**
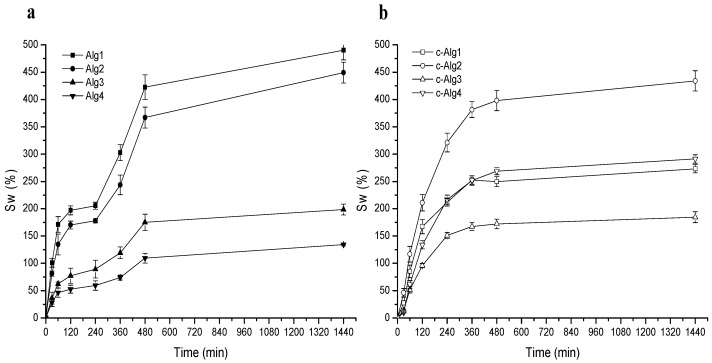
(**a**) Swelling curves of Alg hydrogels; (**b**) swelling curves of c-Alg hydrogels.

The water uptake ability of hydrogels was monitored with time by weight determinations. Swelling kinetics curves are reported in Figure 3a,b. Alg hydrogels (Figure 3a) show a fast water uptake in the first two hours followed by a short plateau and a second phase in which swelling rapidly increases, perhaps due to incoming gel disintegration, until achievement of the equilibrium water content after around 8 h. Swelling is generally related to crosslinking degree: hydrogels with a low crosslinking density are expected to have a large pore size and greater ability to swell, but ultimately may tend to dissolve. On the contrary, an increase of Ca^2+^ concentration or a pH decrease would limit swelling ability. Accordingly, swelling regularly decreases going from Alg1 to Alg4. Alg1 and Alg2 (low calcium content) reach over 400% swelling, whereas Alg3 and Alg4 swell less (below 200%).

Swelling of c-Alg hydrogels increases regularly for all samples (Figure 3b), but the trend is not regular any more: whereas c-Alg2 preserves a high swelling percentage, close to that of Alg2, in the case of c-Alg1, swelling falls from ~460% to ~200% upon coating. This suggests that an alginate hydrogel with a low calcium concentration and besides being characterized by a basic pH, such as Alg1, holds a greater amount of CH-Cl, due to the high number of free carboxylate groups available for ionic interactions with ammonium groups of CH-Cl, thus creating a tight coating layer in which ion pairs are uniformly distributed. It is known from the literature that ionic aggregates represent a barrier to water diffusion [39]; the strong swelling decrease of c-Alg1 is likely a consequence of the reduced water diffusion throughout the coating. Concerning c-Alg2, instead, the partial protonation of carboxylates, as evidenced by the pH value of Alg2, reduces the ionic interactions between alginate and chitosan hydrochloride; thus, CH-Cl is just physically absorbed, and no significant variations of swelling properties are detected. Similarly, in the case of c-Alg3, carboxylate groups are saturated, due to the large excess of calcium ions, and no change of swelling occurs. On the opposite end, c-Alg4 shows higher swelling with respect to Alg4. This result is reasonably related to the re-uptake of water expelled during the coating process as a consequence of shrinkage (see Table 1 and the Discussion Section).

### 2.4. Stability in Normal Saline Solution

**Figure 4:**
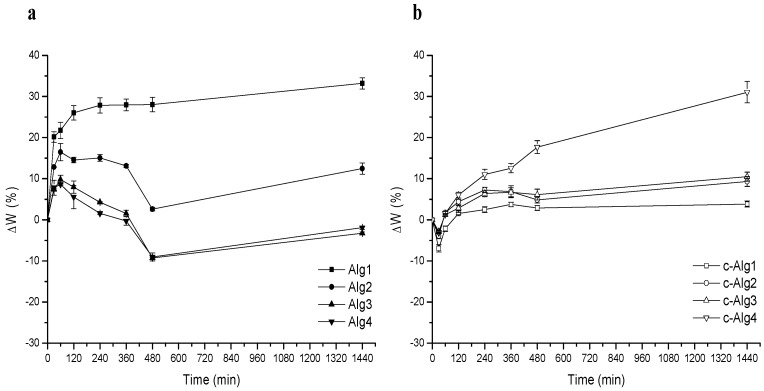
(**a**) Stability test in normal saline solution of Alg hydrogels; (**b**) stability test in normal saline solution of c-Alg hydrogels.

In order to verify the resistance of hydrogels to degradation in a medium close to the environment of the wound bed, a stability test was performed by immersion in normal saline solution for 24 h. Curves relative to weight change percentage with time are shown in Figure 4a,b. It is important to underline that weight loss due to alginate chain dissolution is partially hidden by simultaneous swelling.

From a comparison, it is evident that coating induces an increase of stability: after initial swelling, Alg hydrogels undergo around a 10% weight loss in eight hours, whereas c-Alg does not lose weight during the whole range of time. As can be seen, c-Alg has just a low weight loss of about 5% at first, attributed to the loss of CH-Cl slightly absorbed onto the surface. Interactions between alginate and chitosan hydrochloride are not sensitive to sodium exchange, so the coating is stable, and disintegration of the hydrogel is delayed. It is also worth noticing that the Alg1 and Alg2 samples appear broken into several pieces just after two hours, while all other hydrogels preserve their physical integrity during 24 h.

### 2.5. Cryo-SEM Analysis

**Figure 5:**
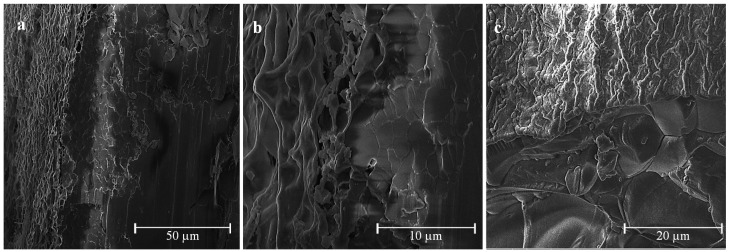

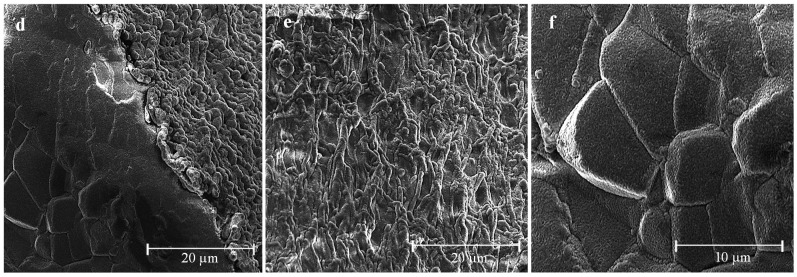
(**a**–**c**) Cryo-SEM images of Alg1; (**d**–**f**) cryo-SEM images of c-Alg1.

Coated and uncoated hydrogels have been observed by cryo-SEM. Micrographs of Alg1 and c-Alg1 are reported in Figure 5a–f as an example. From a comparison between the outer surfaces of Alg1 (Figure 5a,b) and c-Alg1 (Figure 5d,e), it seems that in the last case, the surface is more regular and compact, whereas Alg1 shows an irregular surface, with the presence of numerous depressions, likely due to water evaporation. This evidence suggests a reduction in water loss from the coated hydrogel. Low-temperature fracture allows the exposure of the internal structure; the pore dimension can be roughly estimated in the range of 10–20 μm (Figure 5c,f). The morphology of the internal structure is not altered by the presence of the coating layer.

### 2.6. Release Study

**Figure 6:**
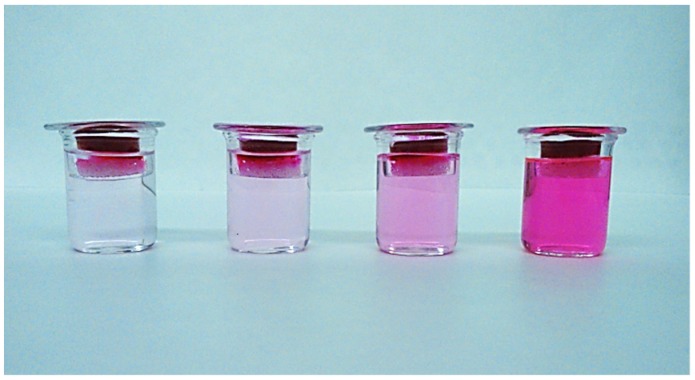
Equipment employed to study the release of rhodamine B in physiological conditions. The photo refers to different release times.

**Figure 7:**
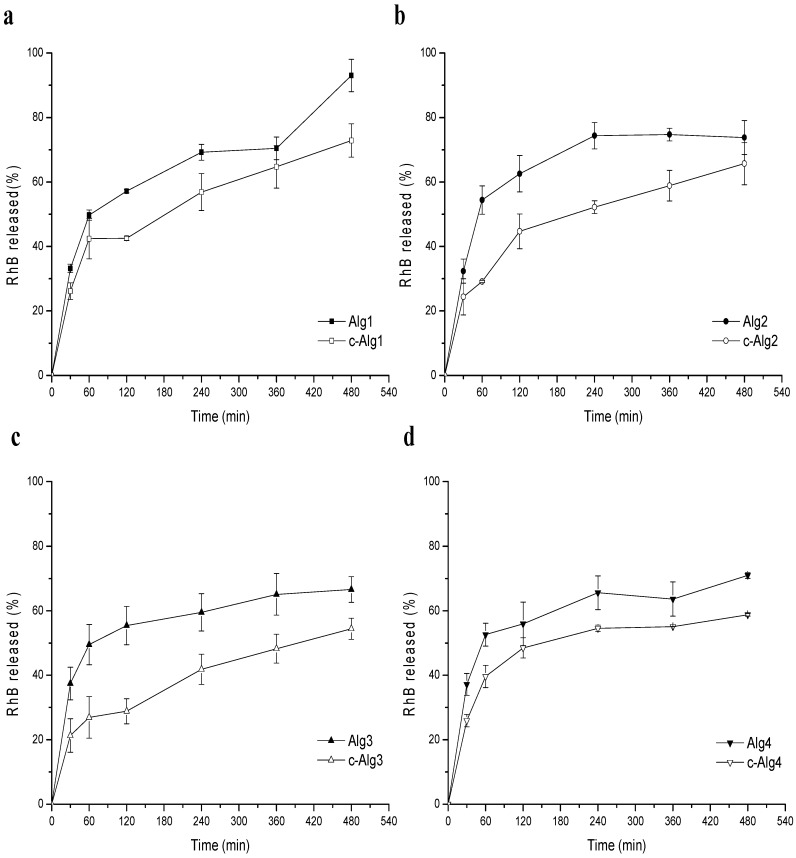
Release profiles of RhB from: (**a**) Alg1 and c-Alg1; (**b**) Alg2 and c-Alg2; (**c**) Alg3 and c-Alg3; (**d**) Alg4 and c-Alg4.

In order to verify the effect of coating on the release properties, rhodamine B (RhB) was chosen as a model of a low molecular weight hydrophilic drug and encapsulated within the hydrogel. To evaluate the release kinetics in physiological conditions, hydrogels were placed on a porous glass set in order to be in contact with PBS medium (pH 7.4) only by the lower surface (Figure 6). This equipment was designed to mimic exudate penetration into the hydrogel from the wound bed. The release profiles of Alg hydrogels compared to coated hydrogels are reported in Figure 7. As can be seen, c-Alg always exhibits a lower release rate with respect to the relative uncoated hydrogels. After eight hours, Alg hydrogels released about 70%–80% of their content, whereas c-Alg hydrogels released only about 60%–50%. The different rate of release can be easily related to the presence of a coating layer on the hydrogel surface. Release from hydrogels is dependent on the diffusion of drug molecules throughout the alginate matrix; the presence of a coating layer acts as a barrier that delays diffusion and, hence, slows down release.

### 2.7. Cytotoxicity Test

**Figure 8:**
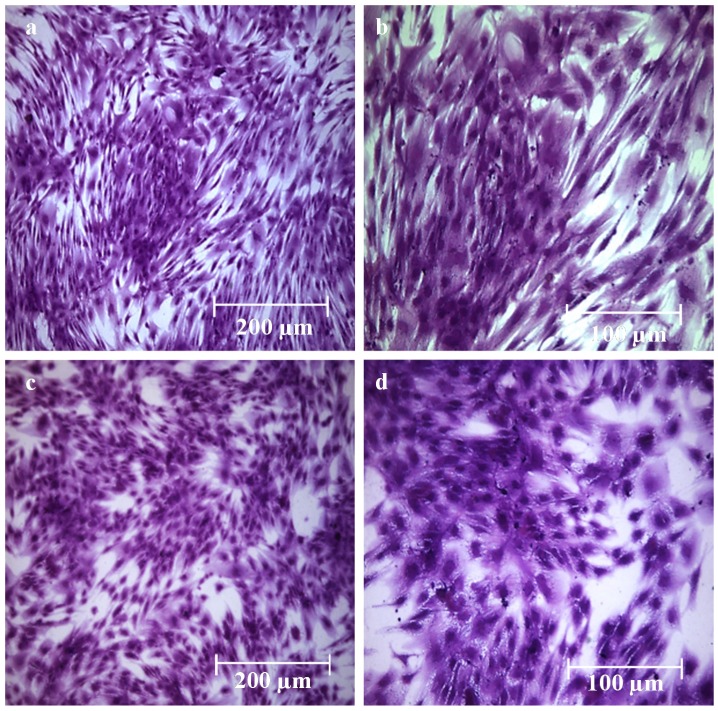
(**a**,**b**) Microscopic images of MSC cultured for seven days in control culture medium after crystal violet staining; (**c**,**d**) microscopic images of MSC cultured for seven days in 0.1% CH-Cl culture medium after crystal violet staining.

A cytotoxicity test to analyze the effects of CH-Cl solution on mesenchymal stromal cells (MSC) was performed. Cells were treated for seven days with culture medium (control) or with culture medium added with 0.1% (*w*/*v*) solution of CH-Cl. MSC were examined every day for one week to observe any possible appearance of cytotoxicity signs, such as morphologic changes, cellular lysis areas or cell death. Figure 8 shows the microscopic images of cells cultured for one week in control medium (a,b) and in 0.1% CH-Cl medium (c,d) after crystal violet staining. It appears evident that in both cases (control and treated MSC), the morphology was substantially unmodified; most important, growth was not inhibited, and cells were able to reach confluence to the same extent as the controls. Anyway, it has to be underlined that this test represents only the first, pivotal approach in the evaluation of biocompatibility, which allows one to exclude an acute cytotoxicity. Other tests, namely sensitization and irritation tests, will be necessary to confirm the absence of adverse effects on living tissues.

### 2.8. Antibacterial Activity

The antibacterial activity of CH-Cl and c-Alg was tested against the *Escherichia coli* wild-type strain, the second most common single pathogen involved in postoperative wound infections [40]. To test the activity of CH-Cl as an antimicrobial agent, MIC and minimum bactericidal concentration (MBC) were determined. MIC and MBC define the antibacterial efficiency of an antimicrobial agent in terms of the concentration at which it will inhibit growth (MIC) or completely kill (MBC) 1 × 10^6^ challenge microorganisms during 24 h of incubation. Both MIC and MBC were found to fall at a 1:32 dilution, corresponding to 0.31 mg/mL of CH-Cl.

**Figure 9:**
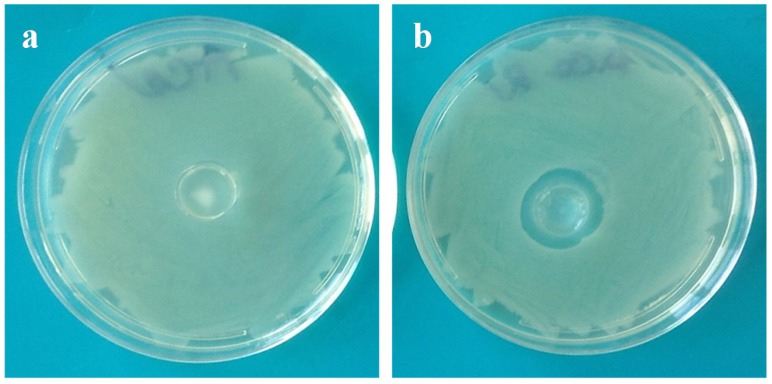
(**a**) Optical photo of *E. coli* growing on agar in contact with Alg1; (**b**) optical photo of *E. coli* growing on agar in contact with c-Alg1.

Preliminary tests based on the solid agar medium contact method carried out on hydrogels evidenced the presence of an inhibition zone around the contact area in the case of coated hydrogels, completely absent in uncoated hydrogels. Figure 9a,b shows photos of *Escherichia coli* growing on agar plates in contact with Alg1 and c-Alg1 hydrogels as an example. No significant differences were noted among the coated hydrogels (c-Alg1, c-Alg2, c-Alg3 and c-Alg4).

**Figure 10:**
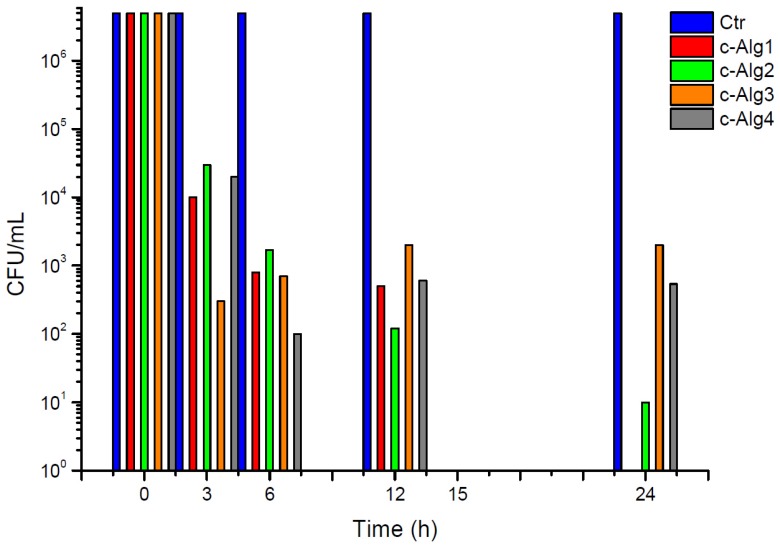
Antimicrobial activity kinetics of c-Alg hydrogels against *E. coli*.

The kinetics of antimicrobial activity against *Escherichia coli* of coated hydrogels is reported in Figure 10. Uncoated hydrogels were tested as the negative control (Ctr). Results show that all c-Alg cause a bacterial inactivation higher than 99% already after 3 h of contact, and a complete killing of bacteria was reached after 24 h in the case of c-Alg1. The concentration of CH-Cl is likely equal or higher than the MIC and MBC values in all coated hydrogels. The slight difference in bactericidal activity among hydrogels is attributed to the different degree of interaction between CH-Cl and alginate in the various hydrogels, as previously discussed (e.g., swelling behavior, see Section 2.2). Alginate hydrogel with the highest concentration of free carboxylate groups (Alg1) binds more CH-Cl via ionic interactions with ammonium groups, forming a denser layer coating on the surface. As a consequence, bacterial killing is complete, whereas surviving bacterial cells (less than 1%) are found with the other hydrogels. Furthermore, in the case of c-Alg3 and c-Alg4, the activity stops after 6 h, as no further reduction is detected after this time. Once again, the occurrence of antibacterial activity can be related to the interaction of alginate with CH-Cl. In fact, in the case of hydrogels with high calcium content (c-Alg3 and c-Alg4), most of the carboxylates groups of alginate are engaged in calcium ion chelation and not available for interactions with ammonium groups, reducing the coating layer.

## 3. Experimental Section

### 3.1. Materials

Pharmaceutical-grade alginic acid sodium salt (Alg), extracted from *Laminaria hyperborean* (viscosity 360 mPa·s, 1% *w*/*v* water solution; mannuronic and guluronic acids: ratio 1.8:2.2), was supplied by Farmalabor (Canosa di Puglia, Brindisi, Italy). Chitosan (CH) extracted from crab shells (high molecular weight; viscosity 400 mPa·s, 1% acetic acid (20 °C); 80% deacetylation degree), calcium carbonate, d-(+)-glucono-δ-lactone (GDL), rhodamine B (RhB) and sodium phosphate monobasic monohydrate (Na_2_HPO_4_·H_2_O) were purchased from Sigma Aldrich (Milan, Italy). Streptomycin sulfate was supplied by Applichem (Darmstadt, Germany). Potassium chloride, sodium bicarbonate, calcium chloride hexahydrate and sodium chloride were purchased from J.T. Baker (Avantor Performance Materials, Milan, Italy). Yeast extract, tryptone and agar, used as the culture medium, were purchased from Oxoid, England. Opti-MEM was purchased from Life technologies Italia (Monza, Italy).

### 3.2. Preparation of Chitosan Hydrochloride

Water-soluble chitosan hydrochloride (CH-Cl) was prepared following literature reports [34]. Briefly, a solution of chitosan (1 g/100 mL, 1% acetic acid) was filtered and dialyzed against NaCl 0.4 mol/L for eight days. NaCl solution was then replaced by freshly-distilled water, and the dialysis continued for another two days. CH-Cl was finally recovered by freeze-drying in the form of white powder.

### 3.3. Preparation of Alginate Hydrogels by Internal Setting Method

Alginate hydrogels were prepared using the internal setting method [35,36]. Fine suspensions of calcium carbonate in distilled water (0.1% or 0.2% *w*/*v*) were obtained by sonication (Vibracell VC 505, Sonics, Newton, CT, USA; 500 Hz, 50% amplitude, 10 min). One gram of sodium alginate was dissolved in 100 mL of suspension under stirring, then a chosen amount of 1% *w*/*v* GDL solution was added. Five milliliters of each final alginate solution were immediately poured into a petri dish (3.6 cm diameter) and left for 24 h in air at room temperature to complete gelation. Hydrogels were washed in water by quick immersion and briefly blotted on paper before characterization.

### 3.4. Preparation of Coated Hydrogels

Samples coated with CH-Cl were prepared by immersion in CH-Cl solution. Freshly-prepared alginate hydrogels (1.9 cm diameter, corresponding to 1 mL of alginate solution) were embedded in 10 mL of CH-Cl solution (1 g/100 mL, water) for 60 min under mild stirring. The coated hydrogels were carefully recovered with a strainer and withdrawn by briefly blotting on paper.

### 3.5. FTIR Analysis

FTIR spectra were obtained in the attenuated total reflection mode (ATR) using a Perkin-Elmer spectrometer (Norwalk, CT, USA) equipped with universal-ATR accessory, fitted with a diamond optical element and ZnSe focusing elements. The apparatus operates with a single reflection at an incident angle of 45°. The analysis was carried out on powders at room temperature and ambient humidity. Spectra were acquired between 4000 and 400 cm^−1^ with a spectral resolution of 4 cm^−1^ and 32 scans collected.

### 3.6. Gelation Time

Gelation time, defined as the time between the addition of GDL and the formation of a fixed gel, was assessed using a method adapted from the literature [38]. The petri dish containing the gelling solution was periodically tilted during the gelation process. Gel was said to be formed when there was no longer flowing when the dish was kept at an angle of 45° for more than 30 s.

### 3.7. pH Measurements

Bulk pH determinations were carried out by a CRISON 507 pH-meter (CRISON, Barcelona, Spain) equipped with type 52-00 electrodes and a probe tip 52-32 for penetration analysis.

### 3.8. Water Content

Water content (W) of hydrogels was calculated as a percentage by the ratio between the wet gel weight and the dry gel weight (after drying at 40 °C in air until constant weight). Measurements were performed in triplicate.

### 3.9. Homogeneity

The homogeneity of alginate gels was estimated according to the literature [38]. Hydrogels were cut into four slices of equal dimension (a–d). Each slice was weighed, then dried to constant weight and reweighed. The dry to wet weight ratio (W_d_/W_w_) of slices provides an indication of the homogeneity. A homogeneous gel will have a consistent dry to wet weight ratio across its constituent slices. Data are the mean of three measurements for each sample.

### 3.10. Swelling

Swelling was determined gravimetrically by monitoring the water up-take with time. Hydrogels were immersed in distilled water, withdrawn at different times and weighed after blotting on paper. For each sample, the measurements were performed in triplicate, and average data were used for the calculations.

### 3.11. Stability Test

A degradation test in normal saline solution (NaCl 0.9%, *w*/*v*) was achieved in order to get information about the stability of hydrogels when in contact with physiological fluids. Hydrogels were weighed and immersed in 100 mL of saline solution at room temperature. At set time intervals, the samples were withdrawn, briefly blotted on filter paper and weighed. Stability was expressed as weight variation percentage (ΔW%). All measurements were performed in triplicate, and average data were reported.

### 3.12. Cryo-SEM Analysis

The morphology of coated hydrogels was examined through cryo-scanning electron microscopy (cryo-SEM) using a cryo-system Gatan Alto 1000E (Gatan, Pleasanton, CA, USA) installed on a FEI Quanta 200 FEG SEM (FEI, Eindhoven, The Netherlands). The sample was placed on the holder, mounted on the cryo-transfer rod, slam-frozen in nitrogen slush and transferred to the cryo-chamber, where it was cryo-fractured and sputter coated with gold/palladium. The sample was finally moved to the SEM chamber where either fracture or top surfaces were observed at −140 °C, using an acceleration voltage of 5–10 kV.

### 3.13. Release Study

Alg and c-Alg hydrogels (1.9 cm diameter), prepared as previously described (Section 2.3), were loaded with RhB by dissolving 10 mg of alginate in 1 mL of RhB aqueous solution of different concentrations, in order to obtain in each gel a final RhB concentration of 13.05 μM. Release studies were performed using a modified Enslin apparatus, designed to mimic the wound bed [41]. Twelve milliliters of phosphate buffer saline solution (PBS; NaCl 120 mM, KCl 2.7 mM, Na_2_HPO_4_ 10 mM, pH 7.4) were in contact with the hydrogels only by their lower surface. At set times, 1.0 mL of buffer medium was withdrawn, replaced by the same amount of fresh PBS and analyzed by UV spectroscopic analysis. Release was followed for 8 h. The RhB% released was calculated using a 10-point calibration curve (*R*^2^ = 0.9993) in the concentration range 13.05–1.30 μM. Experiments were performed in triplicate.

### 3.14. Cytotoxicity Test

In order to exclude the cytotoxic effects of chitosan hydrochloride, an assay was performed on mesenchymal stromal cells (MSC) obtained from normal human bone marrow, as previously described [42]. Cells were seeded in multi-well plates at a density of 20,000/cm^2^ and treated with culture medium (control) or with culture medium added with 0.1% (*w*/*v*) solution of CH-Cl. The test was performed in triplicate. MSC were grown for one week renewing the medium every second day and observed daily by optical microscopy. Finally, control and treated MSC were stained with crystal violet and observed with a Workstation Leica DMI6000 microscope (Leica Microsystems GmbH, Wetzlar, Germany). Images were acquired using a digital camera Leica DFC 340FX (Leica Microsystems GmbH, Wetzlar, Germany) and analyzed by LAS AF 2.2.0 software.

### 3.15. Antibacterial Activity

Antibacterial activity of CH-Cl and c-Alg hydrogels was tested against *Escherichia coli* (DSM 498), purchased from Deutsche Samlug von Mikroorganismen und Zellkulturen GmbH (DSMZ, Braunschweig, Germany). Growth of bacterial strain occurs on sterile Luria-Bertani (LB) medium (10 g/L tryptone, 5 g/L yeast extract, 10 g/L NaCl distilled water) in an aerated incubator at 37 °C, 120 rpm, for 18–24 h. Bacterial growth was verified measuring the optical density of bacteria at 600 nm (OD_600_) by means of a spectrophotometer. After growth and harvesting, bacterial cells were washed twice with sterile Ringer solution (RS), a neutral buffer saline solution (0.150 g KCl, 2.25 g NaCl, 0.05 g NaHCO_3_ and 0.12 g CaCl_2_ per liter of distilled water, pH 7.0) and then re-suspended in RS up to an absorbance of 0.250 ± 0.01, which corresponds to a concentration of approximately 1.5–3.0 × 10^8^ colony-forming units per milliliter (CFU/mL). A second dilution was carried out to obtain working bacterial suspensions of about 10^6^ CFU/mL.

The minimum inhibitory concentration (MIC) of CH-Cl, defined as the lowest concentration of the antimicrobial agent that inhibits the visible growth of the test microorganism, was determined using the broth dilution methods [43,44]. CH-Cl solution (10 mg/mL, water) was serially diluted with LB medium: 10 mL of CH-Cl solution were added to 10 mL of LB and mixed by vortexing for 1 min; successive dilutions were repeated in order to obtain a concentration range spanning from 0.078 to 10 mg/mL. Ten milliliters of each diluted CH-Cl solution were inoculated with 100 μL of 10^8^ CFU/mL microbial suspension of *Escherichia coli*. The inoculum assay and streptomycin sulfate (15 μg/mL) were evaluated as negative and positive controls, respectively; the optical density of bacteria after 24 h was assessed.

The minimum bactericidal concentration (MBC) was measured following MIC determination. One hundred microliters of each of the two dilution suspensions preceding the MIC dilution were plated onto LB medium agar 1.8% and incubated at 37 °C overnight. The highest dilution (and conversely, the lowest concentration) that resulted in a 99.9% reduction in bacterial cells number was recorded as the MBC.

Each measurement was replicated three times.

Preliminary screening of the antimicrobial properties of c-Alg hydrogels (1.9 cm diameter) was carried out by the solid phase contact method on an LB agar medium plate (1.5% agar) in petri dishes. Hydrogels were prepared in sterile conditions. Working bacterial suspensions (0.1 mL) were spread onto the solid surface of the medium agar plate, then hydrogels were put on it, and the plates were incubated at 37 °C for 24 h. After incubation, the inhibition areas were evaluated. Alg hydrogels were tested as the negative control.

The kinetics of killing of c-Alg1, c-Alg2, c-Alg3 and c-Alg4 (1.9 cm diameter) was determined by measuring bacteria logarithmic reduction as a function of time. Hydrogels were kept in contact with 10 mL of working bacterial suspension at room temperature on a wrist-action shaker (ASTM standard Test Method E 2149-01). Then, 0.1 mL of working solution were used to prepare decimal dilutions, which were plated onto the LB solid medium agar plate. Plates were incubated at 37 °C for 24 h. After 0 h (*t*_0_), 3 h (*t*_3_), 6 h (*t*_6_), 12h (*t*_12_) and 24 h (*t*_24_) of contact time, surviving cells were evaluated by the standard plate count method, and inactivation tests were performed in duplicate. The inoculum assay and Alg hydrogels were used as the negative control, while streptomycin sulfate (15 μg/mL) was tested as the positive control. The average colony count of duplicate plates was used to calculate the CFU/mL. Tests were performed three times.

The percentage reduction was calculated by the following equation:

(1) $$\text{Reduction}\;\text{\%}\;\left({\text{CFU mL}}^{-1}\right)=\left(\frac{B-A}{B}\right)\times \;100$$

where *A* = bacterial concentration after a specific contact time and *B* = bacterial concentration at *t*_0_ contact time.

## 4. Conclusions

Alginate chitosan-coated hydrogels were prepared by using water-soluble chitosan hydrochloride. The internal gelation setting method was used for the realization of hydrogels. Coated hydrogels retain good homogeneity and high water content, whereas a decrease of bulk pH was detected. Overall, hydrogels were found to have a water uptake weight percentage ranging from 450 to 200, which would prevent the wound bed from accumulating exudates and, at the same time, protect it from excessive dehydration. Stability in normal saline solution increases upon coating. Cryo-SEM analysis highlighted a more regular and compact surface on coated hydrogels, while the internal morphology was not altered. Coated hydrogels exhibit antibacterial activity against *Escherichia Coli*, and cytotoxicity tests demonstrate that chitosan hydrochloride does not elicit any acute toxic effects on mesenchymal stromal cells. Release studies using rhodamine B as a model of a low molecular weight hydrophilic drug show that the coating induces a decrease in the release kinetics.

To summarize, in this work, novel hydrogels based on alginate and chitosan hydrochloride have been investigated in order to explore their potential application as novel medicated dressings by associating intrinsic antimicrobial activity with improved sustained release characteristics.
